# Development and validation of a rapid assessment tool for malaria prevention

**DOI:** 10.1186/s12936-016-1575-0

**Published:** 2016-11-08

**Authors:** Judith Nekesa Mangeni, Diana Menya, Andrew Obala, Alyssa Platt, Wendy Prudhomme O’Meara

**Affiliations:** 1 College of Health Sciences, Moi University, Eldoret, Kenya; 2 Duke Global Health Institute, Durham, NC USA; 3 Academic Model Providing Access to Healthcare, Eldoret, Kenya; 4 Department of Medicine, Duke University, Durham, NC USA

**Keywords:** Lot Quality Assurance (LQA), Rapid assessment, Problems, Barriers, Malaria prevention

## Abstract

**Background:**

Insecticide-treated bed nets (ITN) have been shown to be efficacious in reducing malaria morbidity and mortality in many regions. Unfortunately in some areas, malaria has persisted despite the scale up of ITNs. Recent reports indicate that human behaviour and mosquito behaviour are potential threats to the efficacy of ITNs. However, these concerns are likely highly heterogeneous even at very small scales. This study aimed at developing, testing and validating a rapid assessment tool to collect actionable information at local levels for a quick evaluation of potential barriers to malaria prevention.

**Methods:**

The study was conducted at the Webuye Health and Demographic Surveillance Site in Bungoma East Sub-County, Kenya. Based on the findings from the case–control study, 12 primary surveillance components that encompass the major impediments to successful prevention were identified and used to develop a rapid assessment tool. Twenty community health volunteers were trained to identify patients with laboratory-confirmed malaria in six peripheral health facilities located within six sub locations and subsequently followed them up to their homes to conduct a rapid assessment. Sampling and analysis of the results of the survey are based on Lot Quality Assurance.

**Results:**

The tool was able to detect local heterogeneity in bed net coverage, bed net use and larval site abundance in the six health facility catchment areas. Nearly all the catchment areas met the action threshold for incomplete household coverage (i.e. not all household members not using a net the previous night) except the peri-urban area. Although the threshold for nets not in good condition was set very high (≥50%), only two catchment areas failed to meet the action threshold. On the indicator for “Net not used every day last week”, half of the areas failed, while for net ownership, only two areas met the action threshold.

**Conclusion:**

The rapid assessment tool was able to detect marked heterogeneity in key indicators for malaria prevention between patients attending health facilities, and can distinguish between priority areas for intervention. There is need to validate it for use in other contexts.

## Background

Insecticide-treated bed nets (ITN) have been shown to be efficacious for reducing malaria morbidity and mortality in controlled trials [1–3]. Therefore, the global health community has invested heavily in ITNs and they have been the cornerstone of global malaria control efforts. More than 400 million ITNs have been distributed in sub-Saharan Africa alone in the last few years [4, 5].

In many places, malaria has declined in response to such efforts [6–8], but success has not been uniform. In some places, malaria has persisted at high levels [9, 10], and in other places malaria has re-emerged after a period of successful control [11, 12]. Current reports have shown the potential threats to ITN efficacy, to include human behaviour [13, 14] and mosquito behaviour [15–17]. However, these problems vary over time and place and are likely highly heterogeneous even at very small scales. There is a need for actionable information collected at local levels to quickly evaluate potential barriers to malaria prevention. Such information could prompt tailored solutions such as improving adherence, targeted ITN distribution to specific groups, or breeding site remediation.

Using data from a detailed case–control study coupled with non-parametric variable selection techniques, a Rapid Assessment Tool was designed to capture information about problems with malaria prevention on a local scale through community health volunteers (CHV). Twenty CHVs were trained to identify patients with laboratory-confirmed malaria infection presenting to peripheral health facilities and to conduct a rapid assessment using the Rapid Assessment Tool during home visits.

The tool was tested in a highly endemic area of western Kenya where ITN coverage is high, but malaria morbidity persists at unacceptable levels. This study has demonstrated that such a tool can be implemented by CHVs, can identify marked heterogeneity in key indicators between neighboring villages, and can distinguish between priority areas for intervention. However, the tool may need refinements which allow it to be deployed using Lot Quality Assurance methodology.

## Methods

### Study area

This study was conducted within the Webuye Health and Demographic Surveillance Site (HDSS), which is located in Webuye Division of Bungoma County, approximately 380 km west of Nairobi. The county borders the Republic of Uganda to the West and lies between latitude 0°25.3′ and 0°53.2′ North and longitude 34° 21.4 and 35° 4′ East of the Greenwich meridian. It covers a land area of 3032 km^2^ or a quarter of the former western province [18].

The main inhabitants of the County are the Luhya ethnic group. Small-scale farming is the main economic activity with sugarcane as the main cash crop. More than 61% of the population lives below the poverty line and majority of the households do not have access to electricity or municipal water. Majority of those in the labour force are mainly engaged in agricultural production which provides 60% of all household incomes; 19% wage employment and 13% urban self-employment [18].

The Webuye HDSS covers approximately 130 km^2^ with an approximate population of 73,000 people in four administrative locations and six sub-locations. Malaria transmission is perennial with a seasonal peak following the rains in May–June. The area is served by Webuye County Hospital as the main referral hospital alongside 12 other peripheral health facilities-mainly health centers and dispensaries.

### Development of the rapid assessment tool

Although malaria control measures have been scaled up in Bungoma East, malaria has not declined in proportion to the magnitude of control efforts. In a recently published case control study, barriers to malaria control that contribute to the persistently high malaria infections in Bungoma East district were identified [19].

Briefly, the case control study measured the efficacy decay of malaria prevention strategies. The main outcome of interest was malaria infection, which indicates that there is a breakdown in prevention. A total of 442 children hospitalized with malaria at the Webuye County hospital were enrolled into the study. They were paired with age, time, village and gender-matched healthy controls in the same community. In addition, comprehensive household and neighborhood assessments including entomological surveillance were done.

### Using bivariate recursive partitioning for variable selection

Variables for inclusion into the rapid assessment tool were selected using bivariate recursive partitioning on a comprehensive set of variables collected in the case control study. Random forests is a non-parametric approach to regression, prediction and variable selection that is particularly well-suited to high dimensional data such as ours where the number of covariates from which to select is large relative to the number of observations [20]. Individual regression trees are constructed via binary splits in numeric predictor variables. The dataset is split into subsets that are increasingly homogeneous with regard to the response variable to form a regression “tree”. Splits are determined by selecting variables with the strongest relationship with the response variable. The process is repeated recursively until a stopping criterion is met. In this case, trees to have may not have nodes with fewer than seven observations. Classification and regression trees have been used in previous studies to rank predictors of malaria illness [21]. However, one conditional inference tree alone may be sensitive to the particular distribution of observations in a dataset leading to overfitting of data, thus the methods of conditional inference trees are expanded by using ensemble methods to create unbiased predictions. First, random subsets of the original dataset are taken repeatedly and conditional inference trees are produced for each subset, in a process called bagging. This reduces the impact of overly influential observations. Second, a subset of predictor variables is taken repeatedly so that conditional inference trees are grown on a subset of both observations and predictor variables.

The relevance of each of the predictor variables in the random forest is summarized by calculating variable permutation accuracy importance. Permutation importance is calculated by summarizing the change in prediction accuracy that occurs when the values of a predictor variable are randomly permuted. All recursive partitioning was done using the party package in R [22]. The top ten predictor variables were selected for inclusion in the rapid assessment tool (variable importance >0.001).

### Using lot quality assurance sample methodology

Sampling and analysis of the results of the rapid assessment are based on Lot Quality Assurance (LQA) methodology. LQA approach requires far fewer observations than traditional sampling approaches [23]. Instead of estimating a population parameter with a pre-specified precision, it tests a sample against a threshold value and determines within a certain confidence whether that sample falls below the critical threshold.

Action thresholds were set by comparing the values of each indicator in the case and control groups. In other words, by comparing the average value of a specific indicator between households with a malaria case and households without a malaria case and choosing a cut-off between those values (Table 1, last column). Exact binomial probabilities for observing D* or fewer failures (f) given a true population prevalence (Pr) of X were calculated for each action threshold X and a sample size of 45. The cutoff value of D* that gave a probability closest to 0.05 without exceeding 0.05 was chosen. In other words the value of D* that satisfies the following:

1 $$ {\text{P }}({\text{f}} \le {\text{D}}^* \, |{\text{ n}} = 4 5,{ \Pr } \ge {\text{X}}) \le 0.0 5, $$

for example, the action threshold for percent of malaria patients who do not sleep under a net every night was set at 30%; if the percent of patients with net non-compliance is at least 30%, then compliance is determined to be a barrier to effective prevention. The value of D* which satisfies Eq 1 is 8 for Pr = 0.3 and sample size of 45. If more than 8 patients are found to be non-compliant in 45, then the null hypothesis of <30% noncompliance is rejected and it is assumed that the population has exceeded the action threshold. There is 95% certainty that the true proportion does not exceed the action threshold if the number of cases does not exceed D* out of n.

**Table 1:**
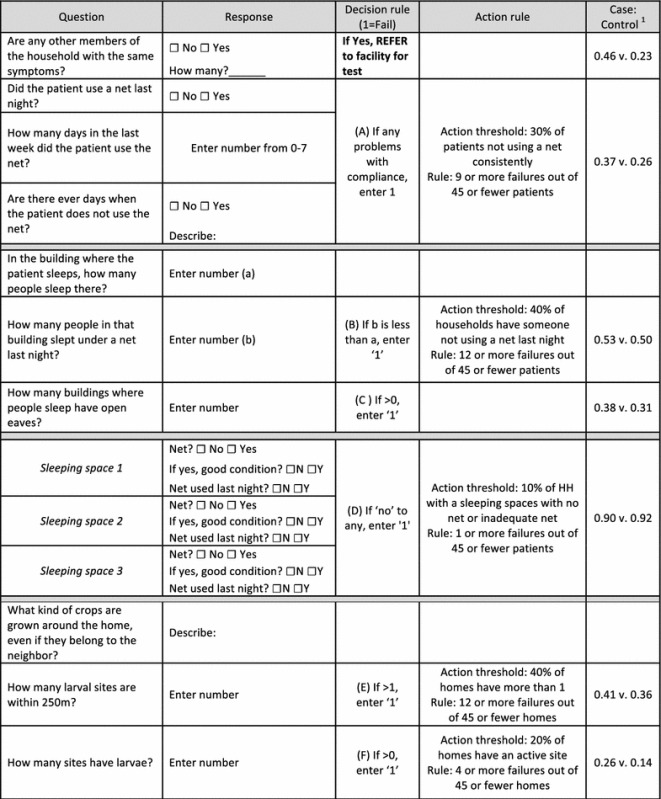
Tool for facility-based assessment of malaria prevention and risk

^1^Proportion of case control children/households passing or failing each indicator. Data are Obala et al. [19]

Table 1 shows the estimated action threshold for each indicator, the critical values D*, and n required to test whether the population exceeds the action threshold with 95% confidence.

After piloting the tool, the indicators and action thresholds were revised. Instead of specifying a sample size of 45 houses, the n and the D* are allowed to vary but pairs of n and D* are identified that would keep the error of misclassifying as acceptable an indicator that exceeded the action threshold X to ≤0.05 (Type I error) and also allowed a maximum type II error (misclassifying an acceptable indicator as unacceptable) of 0.2 when the true prevalence was some lower pre-specified value of Y. In other words:

$$ {\text{P}}\left( {{\text{f }} \le {\text{ D}}^* \, |{\text{ n}},{ \Pr } \ge {\text{ X}}} \right) \, \le \, 0.0 5 $$

$$ {\text{P}}\left( {{\text{f }} \le {\text{ D}}^* \, |{\text{ n}},{ \Pr } \le {\text{ Y}}} \right) \, \le \, 0. 20 $$

the revised n and D* for each indicator along with action thresholds, type I errors, and type II errors for misclassification if the true prevalence is Y are shown in Table 2.

**Table 2:**
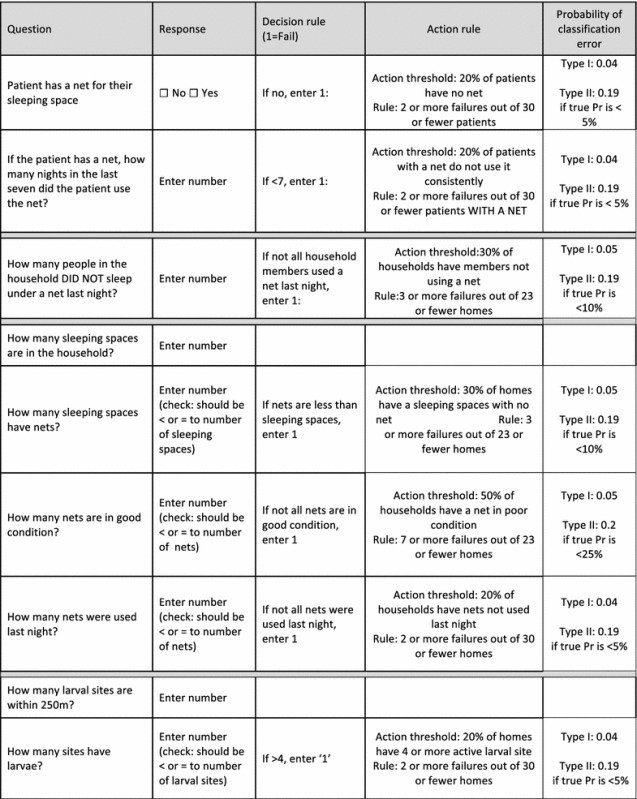
Revised tool for facility-based assessment of malaria risk

### Piloting and assessment of the tool

#### Training and use of the tool by community health volunteers

A total of 20 community health volunteers were trained to use the rapid assessment tool. The training was carried in two parts each consisting of 3–4 h over a period of 2 days. On the first day, community health volunteers were taken through all the items in the rapid assessment tool. The key areas in the training included; checking for other sick family members, identifying larval habitats, and confirming consistent usage of available bed nets. After the training, the CHVs were given an opportunity to do a simulation exercise to ensure the participants had grasped all the areas.

The community health volunteers were given an opportunity to practice the tool the following day in areas that were nearby the Webuye peri-urban center. The research team then reviewed the questionnaires and noted sections where the CHVs had problems. These were given more emphasis to ensure they completely grasped everything. In general, the rapid assessment tool was easily understood and the CHVs administered it with minimal difficulty.

#### Data collection

**Fig. 1:**
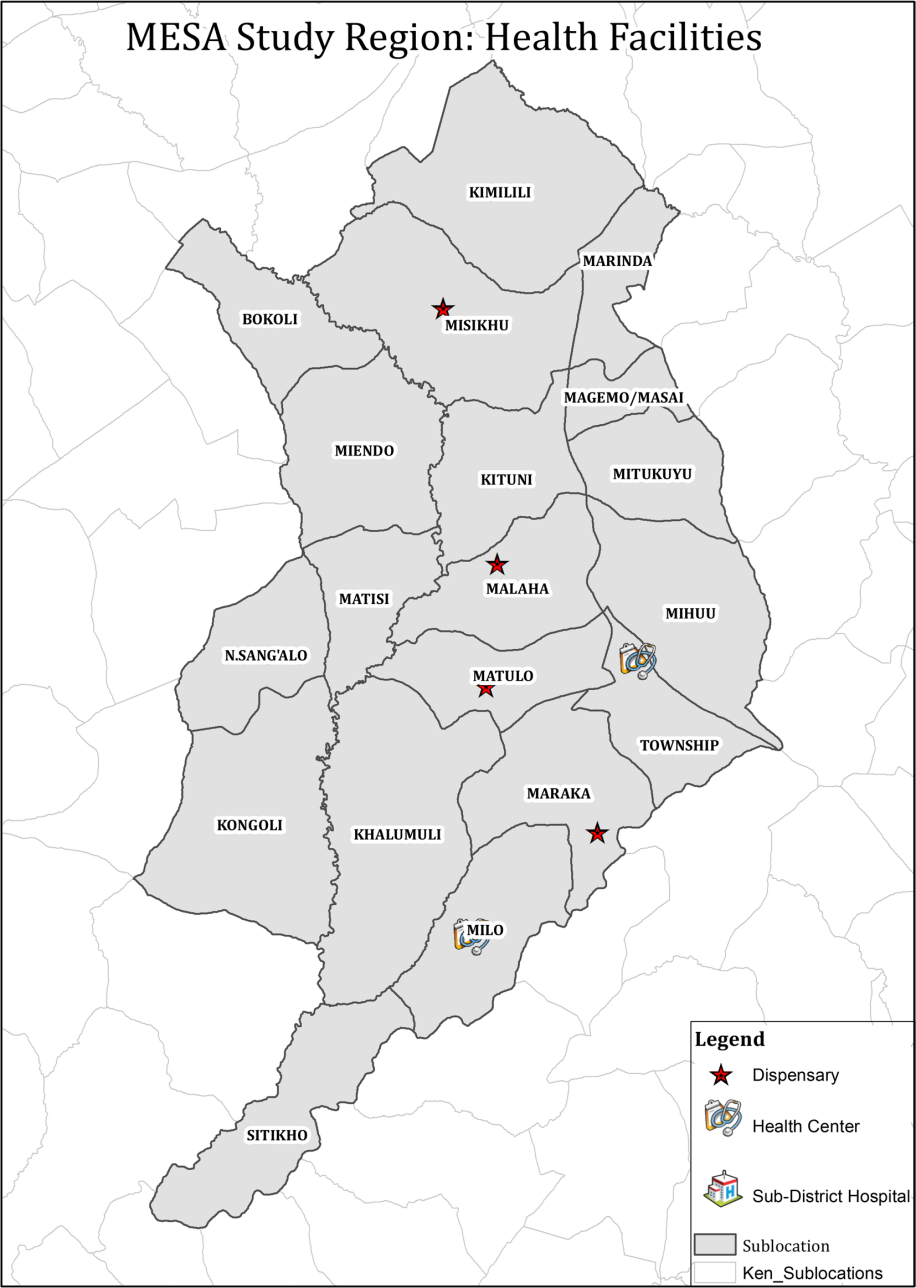
MESA study region: health facilities

The purpose of the tool is to identify problems leading to malaria infection. Therefore, instead of recruiting a random community-based sample, individuals with confirmed malaria infection reporting to the health facility were recruited. There were six peripheral health facilities that were eligible for the study. They had the capacity to perform parasitological confirmation of malaria infection by either microscopy or rapid diagnostic tests and they had CHVs linked to the facility. The six facilities were: Milo, Matulo and Webuye Health Centres, and Lurare, Mukhe and Kayaya Dispensaries, (Fig. 1). Patients with a confirmed malaria infection who were treated as outpatients and were older than one year were recruited consecutively from the laboratory and a CHV accompanied them home to complete the assessment using the tool. Each CHV completed 15 assessments for a total of 45 assessments per facility.

## Results

### Developing the tool using bivariate recursive partitioning

**Fig. 2:**
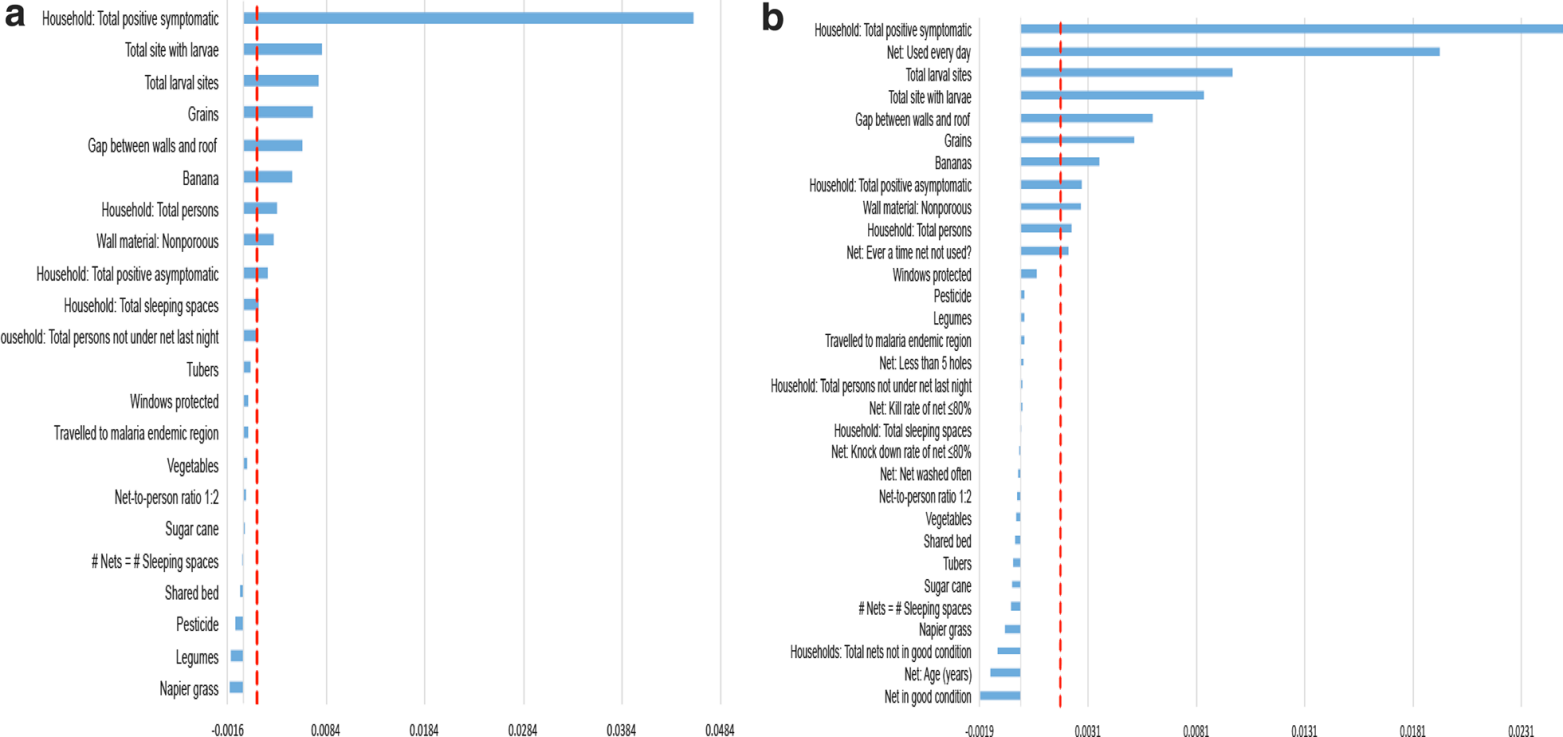
Variable permutation accuracy importance based on bivariate recursive partitioning of the complete set of predictor variables from the case control study. **a** BRP using the entire case–control sample and **b** BRP using only case or control children with a net for their sleeping space

Random forest results are shown in Fig. 2a, b. The variables are ranked in order of importance for (a) the total sample and (b) children who had a net for their sleeping space. For the total sample, selected variables are: total positive symptomatic household members, total larval sites, open eaves, total positive asymptomatic household members, non- porous walls, growing grains, growing banana, neighborhood bed net coverage, neighborhood population density, growing tubers, and total household size. For the subset of patients with nets, the same group of variables were selected from the BRP analysis except ‘growing tubers’ and ‘local bed net coverage’ were no longer above the threshold to be included. The only additional variable was ‘net used every day’.

For this tool, only household indicators were selected and neighborhood variables were excluded. Nonporous walls were also excluded since this was highly correlated with open eaves. It was not feasible to test people for malaria during a rapid assessment, so the participant was asked whether other household members had similar symptoms as a proxy measure.

Selected variables were combined to create the rapid assessment tool. The action thresholds were set based on the difference between these indicators in the case and control children (Table 1). Action thresholds for open eaves and crops grown were not defined since these would be difficult to change, but were included in the tool in order to measure the local heterogeneity in these risk factors.

### Piloting the rapid assessment tool

**Fig. 3:**
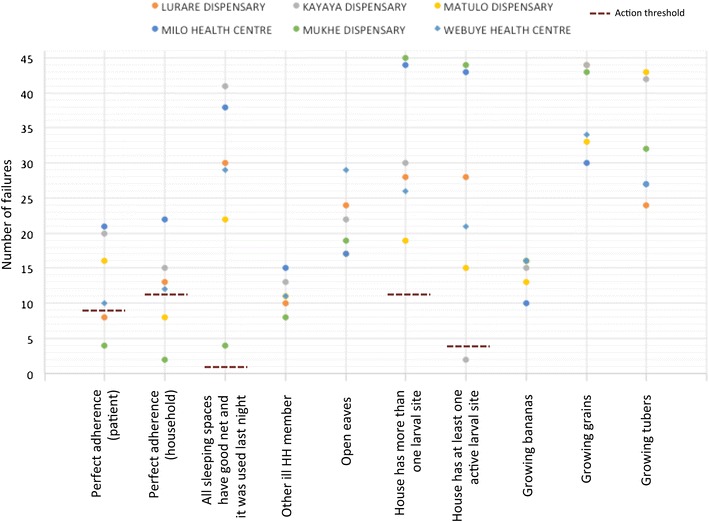
Original rapid assessment tool: number of failures out of 45 by indicator for each health facility. Action thresholds are represented by the *blue dashed line* and facilities exceeding action thresholds are indicated by an *asterisk*

Six peripheral health facilities with capacity to diagnose malaria within the study area were identified and agreed to participate. The average Euclidean distance between facilities is 5.2 km (Fig. 1). 40–46 patients with confirmed malaria infection were recruited from each facility (n = 268) and were visited at home by CHVs using the rapid assessment tool. Recruitment lasted between 1 and 3 weeks in each facility. Each indicator was scored as 0 (pass) or 1 (fail) for each household and the total ‘failures’ were tallied for each health facility. The total failures were compared against the LQA criteria. The number of failures for each indicator, action thresholds, and facilities identified as exceeding action thresholds are shown in Fig. 3.

The tool was able to describe important heterogeneity between geographic areas in each of the indicators. ITN compliance by the patient ranged from 50 to 91%. Near Mukhe dispensary, more than 90% of patients and their households had perfect ITN coverage and adherence, but virtually all homes also had active larval sites nearby. By contrast, less than 5% of homes around Kayaya dispensary had active larval sites nearby, although household ITN coverage and use was very low. Twenty-five percent of households reported another family member ill; the proportion was highest in Milo (32%) and lowest in Mukhe (17%). However, overall the LQA criteria appeared too high; the patient sample from every facility met the action threshold for nearly every indicator. In other words, the tool identified every facility as exceeding the pre-defined action threshold for problems with ITN coverage, ITN use, ITN condition, and larval sites. The tool was better able to isolate areas where coverage was exceptionally good.

### Revising the tool

Based on the results, the tool, sample size, and the action thresholds were revised (Table 2). Importantly, net ownership was separated from net use instead of combining them in the patient compliance indicator. Net condition was also separated from net ownership and net use for each sleeping space. In addition, the minimum sample size and value of D* was identified to achieve a type I error of ≤0.05 (probability of misclassifying a poor performing area as not exceeding the action threshold) and type II error of ≤20% (probability of misclassifying a high performing area as a poor performing area). Type I and II errors are listed in Table 2.

**Fig. 4:**
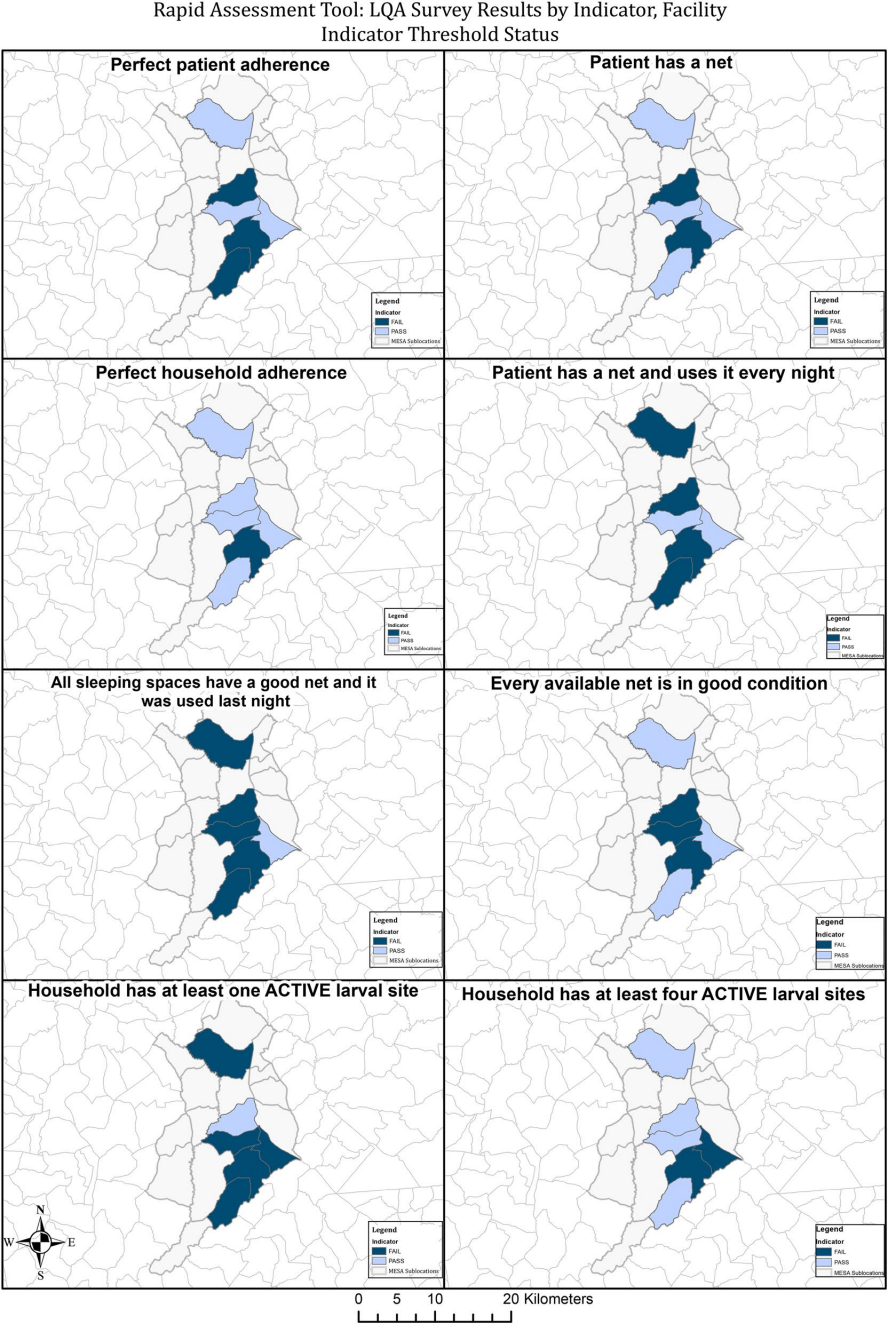
Map of facility catchments in the study area showing which patient catchments passed or failed selected indicators of the revised rapid assessment tool

The data from the 268 patients was then re-analysed based on the new indicators and thresholds. The first 23–30 patients interviewed from each health facility were chronologically identified and this was used as the new sample. The modified approach helped to distinguish between areas where the ‘lot’ of patients did not fail for net ownership, but did fail for net use and vice versa, as well as those facilities which failed for net coverage but available nets were in good condition versus higher coverage but ‘failed’ for net condition (Fig. 4).

The revised tool was able to detect heterogeneity in net ownership, net use, and net condition across the six health facility patient populations. All the six groups met the action threshold for fewer nets than sleeping spaces. Nearly all the groups met the action threshold for incomplete household coverage the previous night except the Webuye Health Centre. Although the highest threshold was for nets not in good condition (≥50%), only two catchment areas (Milo and Webuye Township area) failed to meet the action threshold. On the indicator for “Net not used every day last week”, half of the facilities failed, while for net ownership, only Maraka and Milo met the action threshold (Fig. 4).

## Discussion

As malaria declines in response to control efforts, transmission is becoming more heterogeneous, populations are more susceptible to epidemics, and the need for local information is more acute.

An evidence-based rapid assessment tool that can be implemented at regular intervals (for example, in high transmission areas) or in response to local outbreaks (in low and epidemic prone areas) is presented. Of critical importance, this tool can be deployed by community members with minimal training and little or no external resources.

The tool was designed to employ Lot Quality Assurance sampling which was originally developed for the manufacturing sector, but has been adopted by health programmes and in particular has been used extensively by immunization programmes [24]. Nation-wide survey data has been re-analysed using LQA methodology in order to identify areas with low and high ITN coverage [25]. These analyses have identified notable heterogeneity at a subnational scale, but are only able to give information about the sampled clusters. Only one other study has explored the use of LQA surveys for local malaria surveillance [26]. The approach is much more similar to the way LQA surveys, which are used in polio campaigns to ensure immunization coverage targets are met within small zones in real time [27]. In addition, this study provides a major strength by its prospective nature. No other known studies have used LQA methodology prospectively to identify problems with malaria prevention within a group of diagnosed cases of malaria.

This survey focused on the area immediately surrounding a health facility and used malaria-confirmed cases as the sample. Although this sample is not representative of the entire village, it is representative of people who contract malaria in that village. With a community-based sample areas with different levels of coverage or use could be identified, but it would not be possible to determine whether those differences were responsible for more malaria infection and morbidity. By recruiting confirmed malaria infections, it is possible to link problems with prevention to actual malaria morbidity. In essence, patient populations are compared to see what problems give rise to malaria infections in different areas. Variables were selected for the tool using a non-parametric method that sequentially divides the sample by covariates into groups with different outcomes. This approach was preferred over a regression approach because random forest methods do not assume a functional form or a distribution for sample data. Whereas a regression model assumes additive relationships for predictor variables, a regression tree allows for the possibility of any number of interactions between variables.

Although the tool was developed based on aggregated data from the entire study area, it was able to describe fine-scale heterogeneity in factors that contribute to persistent malaria burden. Communities less than 5 km apart showed distinct patterns—one community had nearly perfect ITN coverage and use but large numbers of larval sites whereas another had very few stagnant water bodies but also low ITN coverage.

The study has several limitations. The zones represented in the study are much smaller than the areas used in polio coverage surveys or the clusters sampled in nationally representative surveys. The indicators used were selected after analysing data from an in-depth case–control study of malaria morbidity in the same area and may not be the optimal indicators for all settings. Furthermore, not all of the variables identified as relating to malaria infection are easily amendable to intervention, such as open eaves and types of crops. In particular, crop types may be related to other environmental factors and were identified as important because they represent proxies for other unmeasured variables.

Such a tool as presented here should promote locally relevant strategies to overcome barriers to prevention. For example, if high prevalence of damaged and old ITNs is identified as the problem, distribution efforts can be scaled up locally perhaps incorporating a ‘trade-in’ strategy. If ITNs are available, but are not being used consistently, then new ITNs are not necessary, but education interventions are required. Regular implementation of the rapid assessment tool can identify emerging problems and prompt early action. In addition, the tool can be used by community members and analysing locally in a very straightforward manner based on targets and rules. This allows the collection, analysis, and subsequent action to be owned by the community.

## Conclusion

Previous malaria rapid assessment approaches have been based on subjective sources such as input from a research team [28], qualitative investigation [29], and in-depth local investigations that involve testing at schools and retrospective data collection [30]. The approach in this study begins with data about risk factors for malaria infection in the region and is, therefore, a stronger and more objective basis for tool development. It is built upon rigorous variable selection techniques using a relatively deep dataset of malaria prevention metrics in an area with a significant burden of malaria. Calibration and adaptation to other contexts would be required.

## Abbreviations

CHVs: Community Health Volunteers
HDSS: Health and Demographic Surveillance Site
ITNs: insecticide treated nets
LQA: Lot Quality Assurance
MESA: Malaria Elimination Scientific Agency
